# TARGETING HEALTH-PROMOTING ACTIVITY IN OLDER ADULTS WITH MULTIPLE CHRONIC CONDITIONS AND FUNCTIONAL LIMITATIONS

**DOI:** 10.1093/geroni/igad104.3616

**Published:** 2023-12-21

**Authors:** Tara Klinedinst, Nicholas Hollman, Carrie Ciro, Darla Kendzor

**Affiliations:** University of Oklahoma Health Sciences Center, Oklahoma City, Oklahoma, United States; OU-TU School of Community Medicine, Tulsa, Oklahoma, United States; University of Oklahoma Health Sciences Center, Oklahoma City, Oklahoma, United States; University of Oklahoma Health Sciences Center, Oklahoma City, Oklahoma, United States

## Abstract

Nearly half of older adults in the U.S. have multiple chronic health conditions in addition to functional limitations (MCC + FL) that restrict health self-management activities. We tested an innovative combination of occupational therapy (OT) and behavioral activation (BA) to improve health self-management using the goal setting, scheduling/monitoring activities, and problem-solving components of the BA approach, as well as skill building, environmental modification, activity adaptation, and focus on daily routines from OT (BA+OT). BA+OT was delivered by occupational therapists over 10 sessions in the homes of participants. We present descriptive statistics from this single-blind randomized control pilot study of 11 older adults with MCC + FL who received BA+OT (n = 6) or enhanced usual care (UC; n = 5). Participants in both groups were given a Fitbit, and the UC group received a handout on managing chronic conditions. The BA+OT group was required to set at least one goal for improving physical activity. On average, participants were 73.1 years old (SD = 8.4), majority female (n = 8), and white (n = 1 Hispanic/Latino). They had an average of 3.9 chronic conditions (SD = 1.5), and 2.0 functional limitations (SD = 1.8). The most common chronic conditions were depression, high blood pressure, and arthritis. Aside from physical activity, the most common participant-identified goal areas were sleep hygiene and positioning, medication routines, and community access. This study is in progress (aiming for n = 40) but shows promise for improving health self-management by targeting meaningful, goal-directed, and health-promoting daily activity.

